# Characteristics of hand injuries caused by corn-husker equipment: a new type of agricultural injury

**DOI:** 10.3389/fsurg.2025.1471142

**Published:** 2025-06-20

**Authors:** Chao-Jian Pang, Xiao-Yan Huo, Yuan Liu, Zong-You Yang, Lu Liu, Xiao-Bo Fan, Shang-Wen Xu

**Affiliations:** ^1^ Department of Orthopaedic Surgery, The First Hospital of Handan, Handan, Hebei, China; ^2^ Department of Orthopaedic Surgery, The Third Hospital of Hebei Medical University, Shijiazhuang, Hebei, China; ^3^ Department of Health Management, Hebei Provincial Hospital of Traditional Chinese Medicine, Shijiazhuang, Hebei, China; ^4^ Department of Hand Surgery, The First Hospital of Handan, Handan, Hebei, China

**Keywords:** hand, agricultural, infection, corn-husker, type

## Abstract

**Objectives:**

This study aims to describe the nature and incidence of hand injuries caused by corn-husker machines, as well as the accident conditions and treatment experiences.

**Methods:**

This retrospective investigation included 34 patients treated at a single institution from August 2018 to August 2021 with postoperative follow-up of more than 12 months. The Hand Injury Severity Scoring System (HISS) was applied to identify the degree of hand injury. Depending on the type of damage, different surgical methods were used, including debridement and suturing, amputation, fracture fixation, negative pressure wound therapy (*N*PWT), skin flap and skin graft procedures, replantation, and tendon repair. Bacterial culture and antibiotic sensitivity tests were performed when wound secretions were present.

**Results:**

The average age was 42.1 years, and 76.4% of the patients were male. 17.6% were classified as HISS I, 20.6% each as HISS II and HISS III, and 41.2% as HISS IV. Among the 83 injured fingers (6 thumbs, 13 index, 12 middle, 10 ring, and 7 little fingers), the finger amputation rate was 57.8%. The most common complication was infection, with 13 cases of postoperative infection, resulting in an infection rate of 38.2%. Although NPWT played an important role in treatment, there was no significant difference in infection rates between wounds treated with NPWT and those treated without. Median hospitalization duration was 35 days (IQR: 27–45); median treatment cost was $4740 (IQR: $3255–$6215). Infections significantly impacted both hospitalization duration and treatment costs.

**Conclusion:**

Corn-husker machine-related hand injuries represent a new type of hand trauma and are often severely mutilating. The selection of appropriate surgical methods and the prevention of infection are crucial aspects in treating such injuries.

## Introduction

Among all economic sectors, agriculture is one of the most hazardous, with a high incidence of accidents, sometimes even fatal, and responsible for occupational diseases (1). Agricultural injuries to the hand and upper extremity are extremely common, representing 40% to 70% of total admissions for injuries that occur on farms (2). Previous studies have shown that upper limb injuries occurring in the farm environment can be very serious and sometimes lead to permanent disability (3). Although hand injuries caused by agricultural equipment are more common in developed countries, with the development of agricultural modernization, such injuries are becoming increasingly prevalent in China. In recent years, with the widespread application of corn-husker equipment in China, severe hand injuries associated with this equipment have also gradually increased (4).

**Figure 1:**
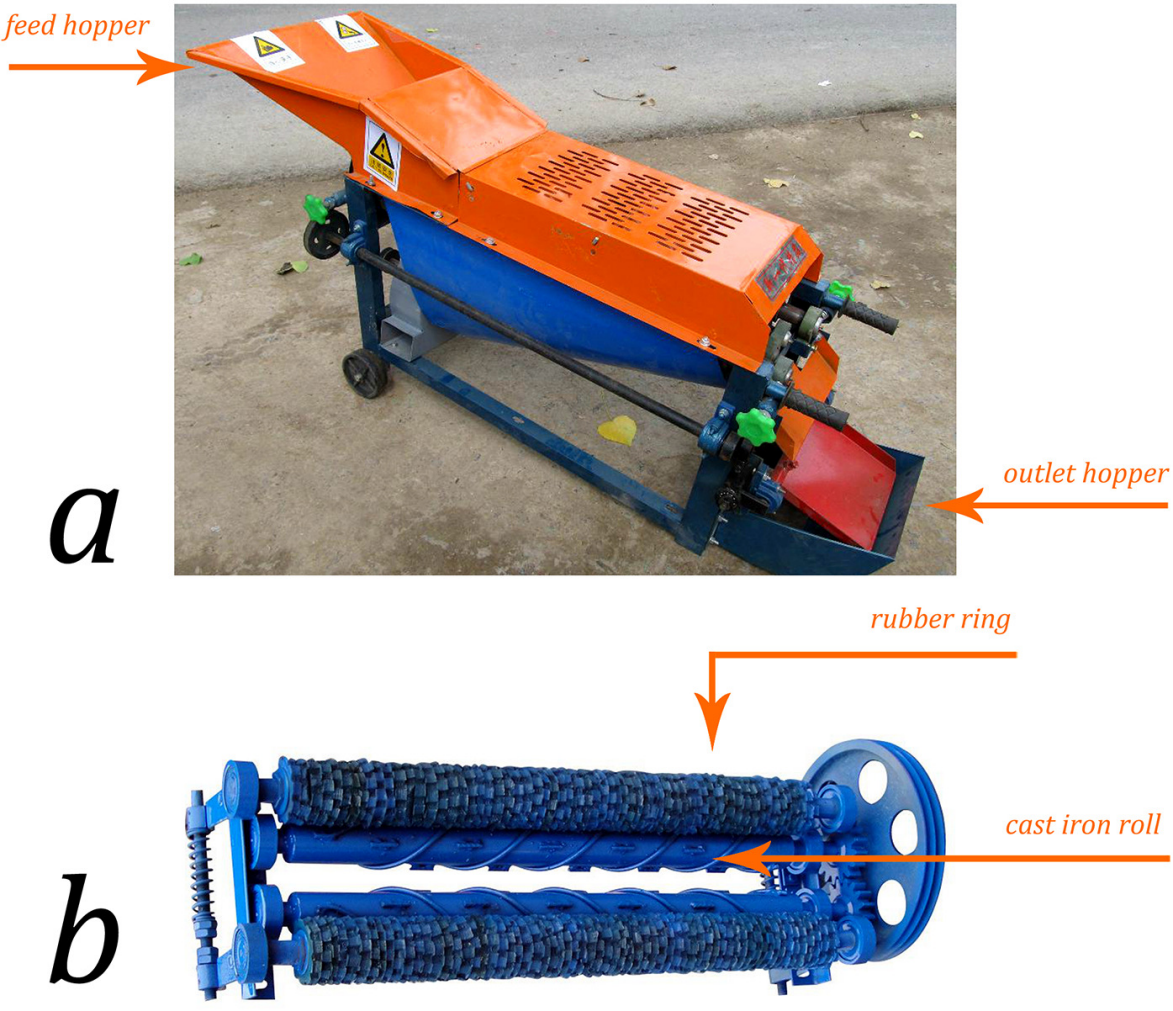
Agricultural machine. **(a)** The main parts of a corn-husker consist of the machine frame, feed hopper, rubber peeling drum, electric motor, husker device, regulating handle, and outlet hopper. **(b)** The end effector of the corn husker is composed of the cast iron roll and rubber ring.

**Figure 2:**
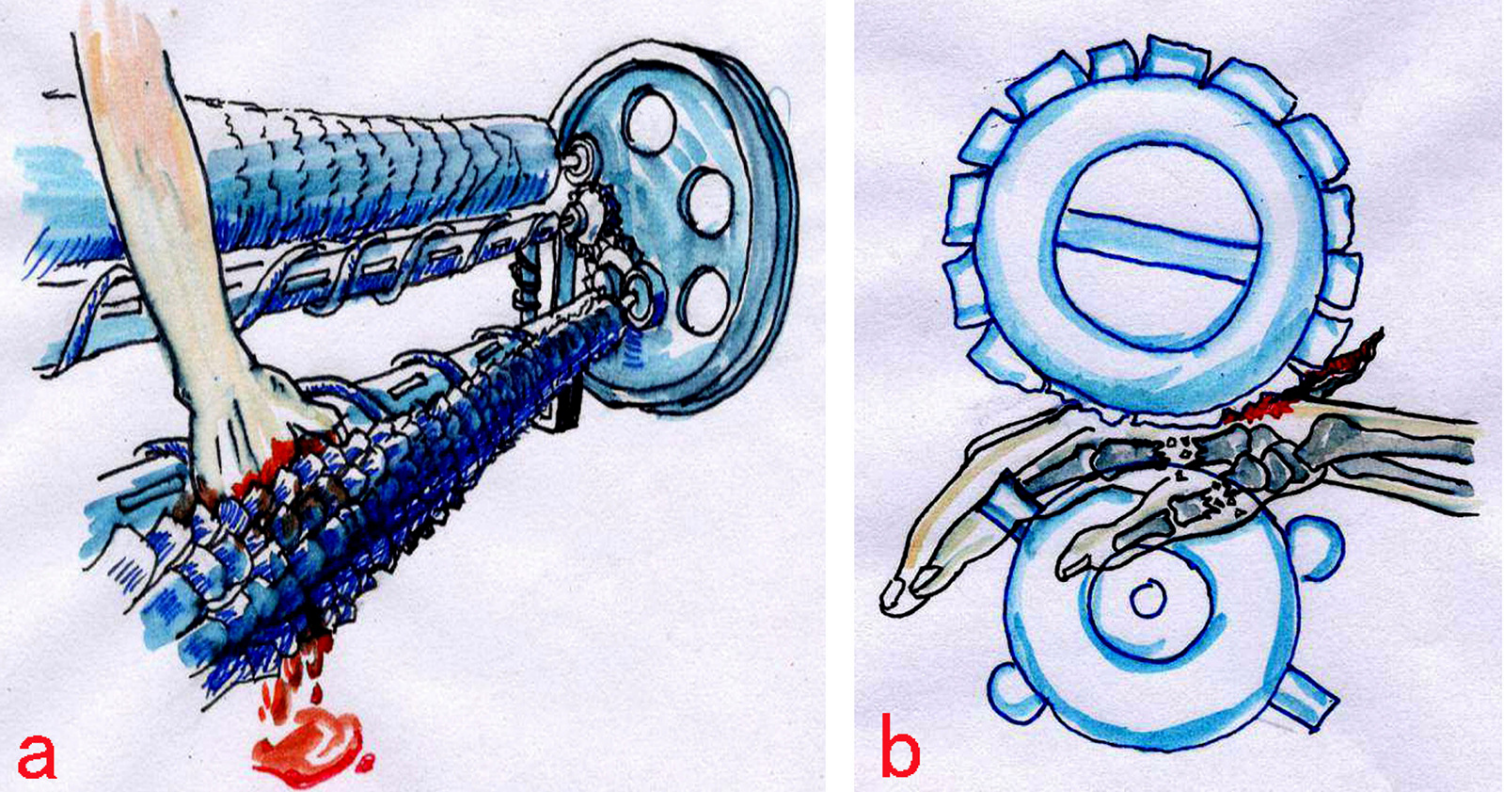
Most accidents resulted from attempts to remove clogged corn by hand while the machines were still operating, leading to hands being caught in the gap between the two rollers.

Corn-husker equipment is commonly used in corn-growing areas in northern China; its main structure is shown in Figure 1. The mechanism of corn-husker injuries often involves operators attempting to remove plugged corn with their hands from the snapping rollers while the machine is running. Momcilovic et al. reported that an operator's hand can be dragged and entangled in a corn picker within 0.15 s, which is twice as fast as the typical reaction time of a healthy person (5). The corn-husker equipment often causes complex injuries to the hands through crushing, tearing, and thermal mechanisms. Therefore, these injuries are usually associated with osseous, soft tissue, neurologic, and vascular damage, resulting in high amputation and infection rates (Figure 2).

## Patients and methods

Approval for this study was obtained from the institutional review board of our institution (W2021-051-1). Inclusion criteria: Acute corn-husker-related hand injuries requiring surgical intervention within 6 h post-injury. Exclusion criteria: Chronic wounds, prior hand surgeries, or incomplete records. Most hand injuries caused by corn-husker equipment are complex traumatic injuries, including metacarpal and phalangeal fractures accompanied by neurologic and vascular injuries. From August 2018 to August 2021, a total of 34 patients with corn-husker equipment-related hand injuries were treated. To evaluate the degree of hand injury, we used the Hand Injury Severity Scoring System (HISS) described by Campbell and Kay in 1996 (6). According to the HISS classification (minor: HISS <20; moderate: HISS 21–50; severe: HISS 51–100; major: HISS >101), the patients were categorized as follows: I (minor), II (moderate), III (severe), and IV (major). Due to the specific injury mechanism and high infection rate, bacterial cultures and antibiotic sensitivity tests were performed on all infected wounds. The study included 26 males and 8 females, with a mean age of 42 years (range: 16–61 years). The mean follow-up period was 12 months.

### Operation methods

In this group of cases, all patients' wounds were open and underwent emergency operations within 6 h after the accident. The basic surgical principles of preventing infection, achieving early healing, and providing optimal function as quickly as possible guided the surgical plan (7). The operation methods included:

Debridement and irrigation: For open wounds, early adequate debridement was performed. The skin around the wound was washed with soapy water, and the wound was irrigated with 0.9% normal saline (NS), dilute iodine, and hydrogen peroxide repeatedly. All nonviable tissues and foreign debris were excised, leaving healthy, viable tissue and protecting vital structures (8). Irrigation performed via gravity using sterile cystoscopy tubing connected to 3-liter bags of saline was preferred over pulse irrigation.

Amputation: Primary amputation was performed when the surgeon believed that adequate wound excision would result in retention of little functional tissue or necessitate late amputation (8). Amputation was carried out for fingers without replantation potential, secondary necrosis after replantation, serious infection, and severely infected fingers.

**Figure 3:**
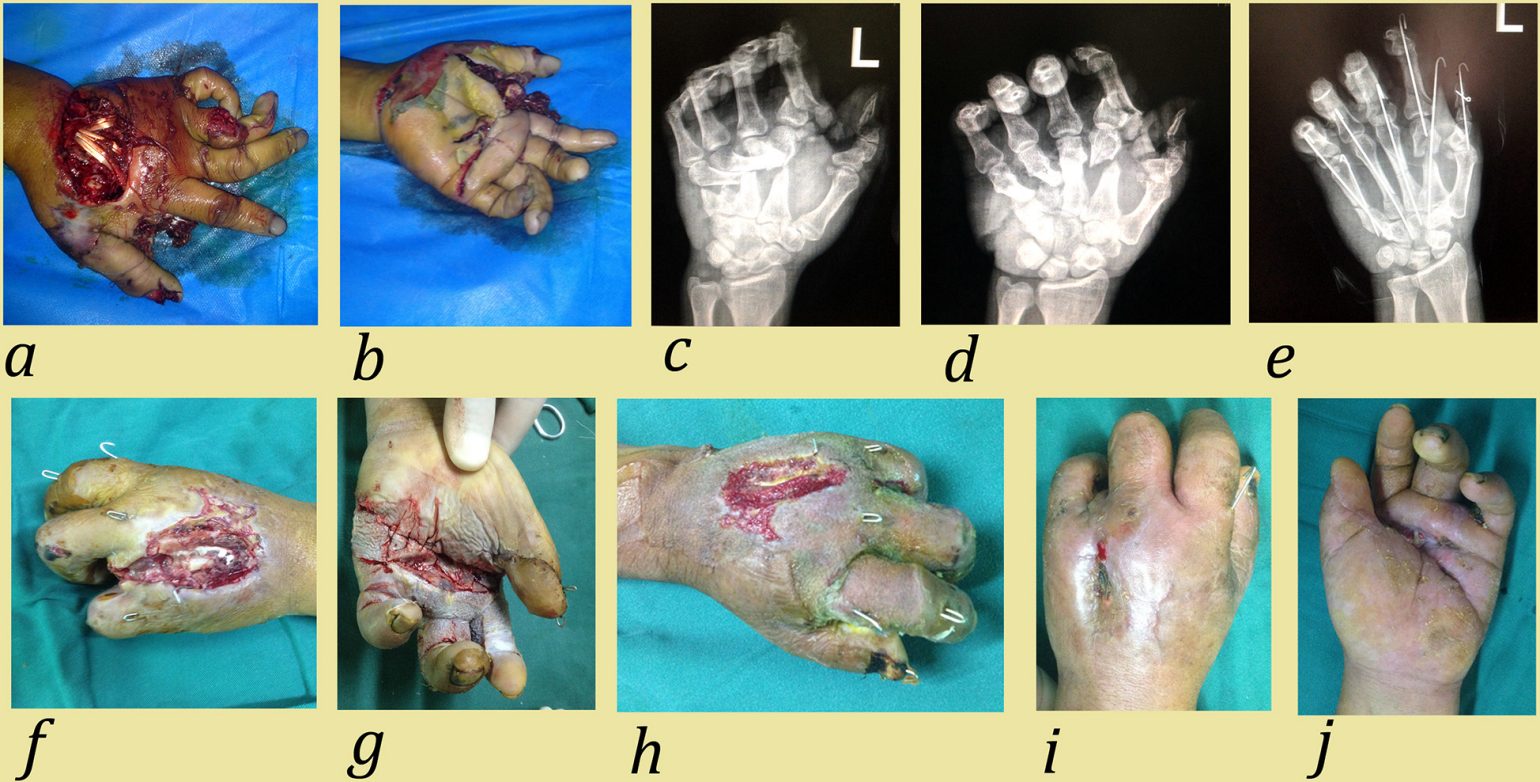
Case 1: A 51-year-old man suffered a corn-husker machine injury to his left hand involving metacarpal fracture, phalangeal fracture, and neurovascular injury. **(a,b)** Preoperative view; **(c,d)** Preoperative anteroposterior and lateral radiographs; **(e)** Postoperative anteroposterior radiograph. Emergency surgical methods included: debridement and suturing, fracture reduction, K-wire fixation, and neurovascular and tendon repair; **(f)** Appearance 3 weeks after the first operation. The necrotic ring finger was removed and NPWT was applied to the wound; **(g,h)** Appearance 4 weeks after the first operation. After NPWT, granulation tissue grew and skin grafting was performed; **(i,j)** Final appearance of left hand. Due to wound infection, complete healing occurred eight weeks after the first operation.

Fracture fixation: In major open injuries, K-wires were quickly placed by retrograde wiring through the fracture site (9). Open fractures and dislocations were common in corn-husker equipment injuries. External fixation and K-wires were used in these injuries. K-wires were inserted in a crossed or longitudinal manner via an antegrade or retrograde approach (Figure 3).

Negative pressure wound therapy (NPWT): For large soft tissue defects and infected wounds, NPWT provided temporary wound coverage. Before NPWT, foreign material, fatty tissues, and devitalized tissues in the wound were completely eliminated. Continuous negative pressure was set at 200 mmHg. The NPWT was removed 3–5 days after surgery. If necrotic tissue, infection, or inadequate growth of granulation tissue was present on the wound surface, NPWT was repeated until the wound was considered clean. The next stage of surgery involved skin flap repair or skin grafting.

Tendon repair: After restoration of reliable vascularity and reestablishment of skeletal stability, all injured tendons were repaired in the early stage. No tendon transplantation or functional reconstruction was performed at this stage.

Replantation: Blood vessel and nerve repair or replantation was performed within 6 h of warm ischemia (without cold ischemia), particularly for large segments. Replanted fingers included 2 thumbs, 1 middle finger, and 1 little finger. All patients were included in the following standardized pharmacological protocol: Fluids: 30–50 cc/kg/24 h; Dextran-40 (molecular weight, 40 kd): 500 cc/24 h; Heparin: 50–100 U/kg/24 h intravenous. This daily pharmacological protocol was carried out for 5 days postoperatively (10).

**Figure 4:**
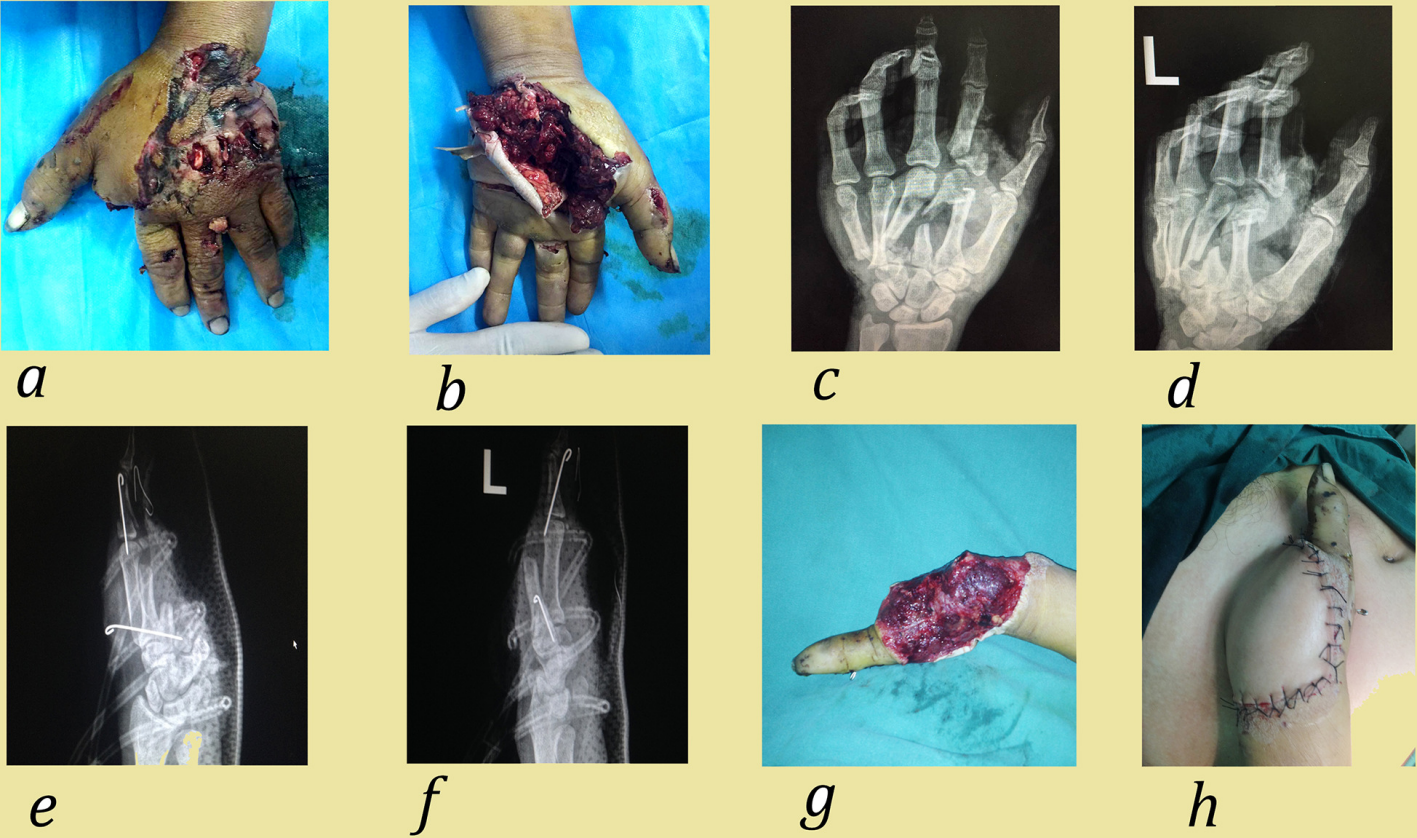
Case 2. A 34-year-old man suffered corn-husker machine injury to his left hand involving multiple metacarpal fractures, neurovascular and tendon injuries. **(a,b)** Preoperative view; **(c,d)** Preoperative positive and lateral radiograph; **(e,f)** Post operative positive and lateral radiograph. The emergency surgical methods include: debridement, fracture reduction, K-wire fixation, amputation (index finger, middle finger, ring finger, little finger and the palmar ulnar side) and NPWT treatment; **(g,h)** Appearance 1 week after the operation. After NPWT, granulation tissue grew and abdominal wall flap was performed.

Skin flap and skin graft: If the damaged area exposed bare bone, tendon, or nerve, or if there was an open joint or bone, it was covered with different types of skin flaps (9). According to the different functional and aesthetic requirements of various hand regions, the flaps included rotation flaps, abdominal wall flaps (Figure 4), peroneal artery perforator flaps, anterolateral thigh perforator flaps (ALTP), or antidromic dorsal interosseous artery island flaps. If the damaged area had a good soft tissue bed, skin defects were covered with split-thickness skin grafts.

### Bacterial culture and use of antibiotics

In open skin injuries, symptoms typically manifest within 10 days after the injury (11), Wound cultures obtained before wound debridement have a very low predictive value and are no longer recommended (12), Hence, infection diagnosis based on the culture of wound swabs was considered more reliable. Bacterial culture and antibiotic sensitivity tests were performed when wound secretions were present.

Early administration of antibiotics is an important factor in reducing the infection rate. Antibiotics were administered 1–2 h prior to the surgical procedure in patients with open wounds, using a combination of cefazolin (2 g every 8 h) + gentamicin (5 mg/kg daily) for 2–3 days. If infection occurred in the wound, the regimen was adjusted based on the identification of specific pathogens. Tetanus prophylaxis followed the Department of Health guidelines (13).

### Data analyses

Statistical analyses were performed using SPSS software (version 26.0, IBM Corp., Armonk, NY, USA). Descriptive statistics were used to summarize patient demographics, injury characteristics, and treatment outcomes. Categorical variables were presented as frequencies and percentages, while continuous variables were expressed as means with ranges or standard deviations.

Fisher's exact test was used to assess differences in infection rates between wounds treated with and without NPWT and to compare hospitalization duration and treatment costs between infected and non-infected patients, as well as between patients with primary and secondary infections. Multivariate logistic regression adjusted for HISS classification was used to control confounding factors. A *post-hoc* power analysis was conducted using G-Power software, based on the observed effect sizes and sample sizes. The analysis indicated high statistical power (1 − *β* = 0.99) to detect significant differences in both average hospitalization duration and treatment costs between infected and non-infected patients. In contrast, the power to detect a difference in infection rates between NPWT and non-NPWT groups was low (1 − *β* = 0.27). Notably, the nonsignificant *p*-value for infection rate comparisons (*p* = 0.481) aligns with this low power. For all statistical analyses, a *p*-value < 0.05 was considered statistically significant.

## Results

**Figure 5:**
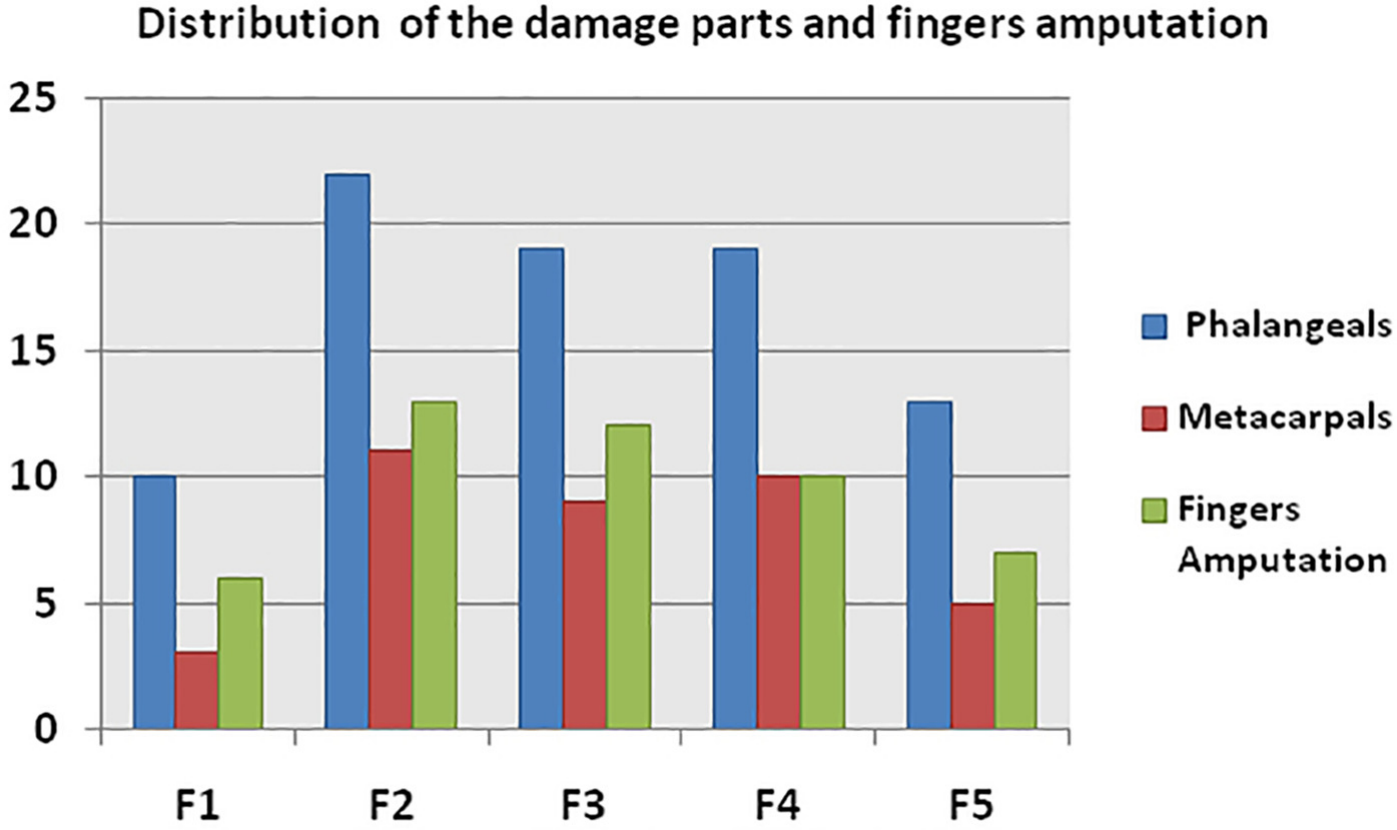
Number of injured fingers, metacarpals, and amputated fingers. F (finger): F1: thumb; F2: index; F3: middle; F4: ring; F5: little finger.

Among the injured parts, there were 17 right hands and 17 left hands, involving 38 metacarpals and 83 phalanges. The 83 injured phalanges consisted of: 10 thumbs (12.0%), 22 index fingers (26.5%), 19 middle fingers (22.9%), 19 ring fingers (22.9%), and 13 little fingers (15.7%). The injured metacarpals included 3 first metacarpal bones (7.8%), 11 s metacarpal bones (28.9%), 9 third metacarpal bones (23.7%), 10 fourth metacarpal bones (26.3%), and 5 fifth metacarpal bones (13.1%). Of the 83 injured fingers, a total of 48 finger amputations were performed, including 6 thumbs, 13 index fingers, 12 middle fingers, 10 ring fingers, and 7 little fingers, resulting in an amputation rate of 57.8% (Figure 5). Two fingers (1 thumb and 1 middle finger) underwent re-amputation due to necrosis after replantation, and four fingers due to severe infection. There were five finger amputations in 1 case, four finger amputations in 4 cases, and three finger amputations in 3 cases.

According to the HISS classification, 14 patients (41.2%) suffered from HISS IV injury, 7 patients (20.6%) each had HISS II and HISS III injuries, and only 6 patients (17.6%) had HISS I injuries (Table 1). Among these patients, 17 cases (50%) received NPWT, with 6 cases requiring NPWT twice due to wound deterioration. Due to soft tissue defects accompanied by exposure of bone, tendon, or neurovascular bundles, various skin flaps were used, including rotation flaps in 2 cases, abdominal wall flaps in 5 cases, anterolateral thigh perforator flap (ALTP) in 1 case, peroneal artery perforator flap in 1 case, and antidromic dorsal interosseous artery island flap in 1 case. All skin flaps were performed in two stages after infection was eliminated, with no postoperative skin flap necrosis. Fifteen cases received split-thickness skin grafts after NPWT, with a 100% survival rate at 2 weeks post-surgery (Table 2). HISS IV injuries accounted for a large proportion (41.2%) of cases, resulting in higher rates of NPWT use (64.7%), flap repair (50.0%), and skin grafting (60.0%) compared to other HISS classifications. Digital replantation was attempted on 4 severed fingers, but 2 fingers developed secondary necrosis due to thrombosis within 5 days post-operation, indicating that the high-energy injury mechanism caused severe vascular damage, leading to a decreased success rate of replantation surgery.

**Table 1:** Demographic and clinical characteristics of patients with corn-husker equipment hand injuries.

Variable	*n* (%)
*N*	34
Gender	
Male	26 (76.5%)
Female	8 (23.5%)
Age (years)	
Mean (range)	42 (16–61)
Injury side	
Right hand	17 (50%)
Left hand	17 (50%)
Number of injured fingers	83
Thumb	10 (12.0%)
Index finger	22 (26.5%)
Middle finger	19 (22.9%)
Ring finger	19 (22.9%)
Little finger	13 (15.7%)
Number of injured metacarpals	38
1st metacarpal	3 (7.8%)
2nd metacarpal	11 (28.9%)
3rd metacarpal	9 (23.7%)
4th metacarpal	10 (26.3%)
5th metacarpal	5 (13.1%)
HISS classification	
Minor (HISS I)	6 (17.6%)
Moderate (HISS II)	7 (20.6%)
Severe (HISS III)	7 (20.6%)
Major (HISS IV)	14 (41.2%)

**Table 2:** According to HISS classification, the different proportion of using NPWT, flap repairment and skin graft.

Treatment	I	II	III	IV
HISS (%)	6 (17.6%)	7 (20.6%)	7 (20.6%)	14 (41.2%)
NPWT (%)	0 (0.0%)	3 (17.6%)	3 (17.6%)	11 (64.7%)
Flap repairment (%)	1 (10.0%)	2 (20.0%)	2 (20.0%)	5 (50.0%)
Skin graft (%)	0 (0.0%)	3 (20.0%)	3 (20.0%)	9 (60.0%)

Bacterial culture and antibiotic susceptibility tests were conducted on pus and wound secretions. Thirteen cases developed postoperative infections, including 6 cases of secondary infections (II: 1, III: 3, IV: 2), resulting in an overall infection rate of 38.2%. Most infected cases (61.5%) were HISS IV injuries, 23.1% were HISS III, 15.4% were HISS II, and no infections were found in HISS I injuries. Among NPWT patients, 8 (47.1%) had positive wound cultures. After adjusting for HISS severity via multivariate logistic regression, NPWT use showed no significant association with infection risk (adjusted OR = 1.4, 95% CI: 0.3–6.1, *p* = 0.621). Fisher's exact test confirmed no statistical difference (*p* = 0.481). (Table 3).

**Table 3:** Infection rate of wound infection in the NPWT and No NPWT groups.

Treatment	Infection	No-infection
NPWT	8 (47.1%)	9 (52.9%)
No NPWT	5 (29.4%)	12 (70.6%)
Total	13 (38.2%)	21 (61.8%)

Note: No significant difference between the NPWT group and no NPWT therapy group (*P* > 0.05).

The isolated pathogenic bacteria in our research included Pseudomonas aeruginosa (5 cases) and Burkholderia cepacia (5 cases), followed by Citrobacter freundii (2 cases), Klebsiella pneumoniae (2 cases), and others including Proteus mirabilis, Staphylococcus saprophyticus, Acinetobacter baumannii, Staphylococcus aureus, and Escherichia coli (Table 4). The number of isolated pathogens varied according to HISS classification, with no pathogens detected in HISS I injuries, but 6 different types of pathogens detected in HISS IV injuries. In all cases, Gram-negative bacilli (GNB) accounted for 89.5% of isolated pathogens, while Gram-positive bacilli accounted for 10.5%. Gram-positive bacilli included Staphylococcus aureus and Staphylococcus saprophyticus. S. aureus was resistant to tetracyclines, macrolides, and clindamycin. S. saprophyticus was resistant to penicillin, clindamycin, and second-, third-, and fourth-generation cephalosporins. Among the GNB, the most common pathogens were Pseudomonas aeruginosa and Burkholderia cepacia. The wounds were often grossly contaminated, and some of the *in situ* bacteria were antibiotic-resistant (14). Among these isolated pathogens, P. aeruginosa, K. pneumoniae, A. baumannii, and E. coli showed antibiotic resistance (Table 5).

**Table 4:** Bacterial culture and drug susceptibility test results of the infected wound.

Bacterial culture result	Cases	Drug susceptibility test results
Pseudomonas aeruginosa	5 (35.7%)	Amikacin, Aztreonam,
		Ciprofloxacin, Gentamycin
Burkholderia cepacia	5 (35.7%)	Meropenem, Ciprofloxacin, Gentamicin
Citrobacter freundii	2 (14.3%)	Amikacin, Ciprofloxacin, Gentamicin, Levofloxacin, Imipenem
Klebsiella pneumoniae	2 (14.3%)	Amikacin
Proteus mirabilis	1 (7.1%)	Ceftazidime, Cefoperazone,
		Cefotaxime Oxime
Staphylococcus saprophyticus	1 (7.1%)	Azithromycin and Doxycycline, co-trimoxazole, Gentamicin, Levofloxacin, Tetracycline, Vancomycin
Acinetobacter baumannii	1 (7.1%)	Amikacin, Ciprofloxacin,
		Ceftazidime, Cefoperazone
Staphylococcus aureus	1 (7.1%)	Amikacin, Ceftazidime, Cciprofloxacin, Doxycycline, Gentamicin, Tetracycline, Vancomycin
Escherichia coli	1 (7.1%)	co-trimoxazole, Imipenem, Cefoxitin

Note: There were 5 cases had a bacterial mutation in the second bacterial culture.

**Table 5:** Number of isolated pathogens and resistance according to HISS classification.

HISS	Isolated pathogens	Distribution of pathogen drug resistance (n)																		
		PIP	TZP	CAZ	CFP	IMP	AZT	LVX	MEC	GM	EM	CD	TC	CFX	CHL	TM	SXT	CX	AM	CFT
Ⅰ	No	–	–	–	–	–	–	–	–	–	–	–	–	–	–	–	–	–	–	–
Ⅱ	Staphylococcus aureus	R	–	–	–	–	–	–	–	–	R	R	R	–	–	–	–	–	–	–
	Pseudomonas aeruginosa	R	–	R	R	–	–	–	–	–	–	–	–	–	–	–	–	–	–	–
Ⅲ	Proteus mirabilis	–	–	R	–	–	–	R	–	–	–	–	–	R	R	R	–	–	–	–
	Pseudomonas aeruginosa	–	–	R	R	–	R	–	–	–	–	–	–	–	–	R	–	–	–	–
	Klebsiella pneumoniae	R	R	–	–	R	–	–	–	R	–	–	–	R	–	R	–	R	–	–
	Acinetobacter baumannii	–	–	–	–	–	–	–	–	–	–	–	–	–	–	–	R	–	–	–
Ⅳ	Klebsiella pneumoniae	R	–	–	–	–	–	–	–	–	–	–	–	R	–	R	–	R	–	–
	Escherichia coli	–	–	–	R	–	–	R	–	R	–	–	–	R	–	–	–	R	R	R
	Citrobacter freundii	–	–	–	–	–	–	–	–	R	–	–	–	R (*n* = 2)	–	–	R	–	R (*n* = 2)	R (*n* = 2)
	Pseudomonas aeruginosa	–	–	R (*n* = 3)	R (*n* = 3)	–	–	R (*n* = 2)	–	–	–	–	–	–	–	R	–	–	–	–
	Staphylococcus saprophyticus	R	–	R	R	–	–	–	–	–	–	R	–	–	–	–	–	–	–	R
	Burkholderia cepacia	–	–	R (*n* = 4)	–	–	–	R (*n* = 3)	R	–	–	–	–	–	R	–	R (*n* = 4)	–	–	–

AM, ampicillin; AZT, aztreonam; CD, clindamycin; CFX, cefuroxime; CAZ, ceftazidime; CFP, cefepime; CFT, cefotaxime; CX, cefoxitin; CIP, ciprofloxacin; CHL, chloramphenicol; EM, Erythromycin; GM, gentamicin; LVX, levofloxacin; IMP, imipenem; PIP, piperacillin; SXT, co-trimoxazole; TZP, piperacillin-tazobactam; TC, tetracycline; TM, tobramycin; MEC, meropenem; R, resistant.

Median hospitalization duration was 35 days (IQR: 27–45); median treatment cost was $4740 (IQR: $3255–$6215). Infected patients had significantly longer stays (median 42 vs. 29 days, *p* < 0.001) and higher costs (median $6421 vs. $3585, *p* < 0.001).

### Long-Term functional outcomes

To assess the long-term functional outcomes, we evaluated the Disability of the Arm, Shoulder, and Hand (DASH) scores and return-to-work rates during the 12-month follow-up. The mean DASH score was 30.2 (range: 14–41). Patients with HISS I and HISS II injuries exhibited lower DASH scores (mean: 16.7 and 24.8, respectively), reflecting better functional recovery. In contrast, patients with severe (HISS III) and major (HISS IV) injuries had higher DASH scores (mean: 32.5 and 37.3, respectively), indicating significant residual disability. Return-to-work rates were also correlated with injury severity. Overall, 85.3% (29/34) of patients resumed work within 12 months. All patients with HISS I and II injuries returned to work, while the return rate decreased to 78.6% (11/14) for HISS IV injuries.

## Discussion

In the last decade, corn-husker equipment-related hand injuries have been increasing gradually in China. Farm machinery is involved in a significant percentage of non-fatal injuries that can cause serious permanent disability (15). This type of injury occurs through various mechanisms, including crushing, tearing, and thermal damage. Seasonal variation in the incidence of injuries is noteworthy, with all injuries occurring from September to December, corresponding to the most active months of corn harvest in northern China (16). The age group that sustained most injuries was between 16 and 61 years, with males predominating; the male to female ratio was 3.25:1.

Characteristic injuries include grossly contaminated lacerations, crush injuries with multiple fractures, severe soft-tissue tears, and primary digit amputation. In this retrospective study, we found that the index finger and the second metacarpal bone were the most commonly injured. Multiple injured fingers were observed in 18 cases (52.9%), multiple injured metacarpals in 10 cases (29.4%), and fingers accompanied by metacarpal injuries in 9 cases (26.5%) (Table 1). These results stem from high-energy trauma involving the bones.

After debridement and irrigation, the surgeon must decide whether reconstruction of digits is viable or if function would be best improved with early amputation and possible future reconstruction (8). Of the 83 injured fingers, a total of 48 fingers were amputated, resulting in an amputation rate of 57.8%. As shown in Figure 5, the number of amputated fingers in HISS IV injuries was the largest, leading us to conclude that the extent of injury is closely related to the occurrence of finger amputation. For devastating injuries, studies have reported that early amputation should be viewed as an appropriate first step toward rehabilitation, rather than a surgical failure (8).

The force of high-energy trauma acts on the incompressible blood in the vasculature, leading to a dramatic rise in tissue pressures and damage to multiple tissue types, including bones, blood vessels, nerves, and soft tissues (17). Due to the damage characteristics of corn husker injuries, a variety of surgical methods were performed, such as debridement, amputation, fracture fixation, NPWT, skin flap and skin graft procedures, replantation, and tendon repairs. Four fingers underwent replantation, but two were amputated at 5 and 7 days postoperatively, resulting in a replantation survival rate of 50%. Based on our experience, we consider arterial wall injury and thrombosis as the main reasons for replantation failure.

NPWT may play a role in the initial management of high-energy, highly contaminated injuries caused by agricultural machinery (18). In corn husker injury cases, skin lesions, necrosis, and defects were common. NPWT has become an important temporary tool that offers a “bridge” in treatment until definitive surgery can proceed safely (19). NPWT offers numerous advantages, including reduced time to attain primary closure of contaminated wounds, decreased incidence of soft-tissue infection, shorter inpatient hospital stays, fewer labor demands, and improved patient comfort (20). In this study, 17 cases (50%) utilized NPWT, among which 6 cases required NPWT twice due to wound deterioration. After NPWT, flaps or free skin grafts were chosen to cover the wound according to the condition of the soft tissue bed. If the wound area exposed bare bone, cartilage, tendon, or nerve, or if there was an open joint, it had to be covered with a flap. In other cases, a split-thickness skin graft sufficed (9). Early skin coverage alleviates pain, prevents infection, and reduces the number of surgical procedures and treatment costs (21).

Among our patients, flaps were chosen in 10 cases to cover the wound, with 50% being HISS IV injuries, according to the nature of the bed and future reconstructive needs. Five types of flaps were selected: rotation flap, abdominal wall flap, ALTP, peroneal artery perforator flap, and antidromic dorsal interosseous artery island flap. Free tissue transfers are relatively complex and time-consuming, requiring microsurgical expertise and often associated with donor-site morbidity (22). Pedicled flaps are not popular due to their disadvantages, such as patient discomfort, bulkiness, and the need for multiple stages. However, among these flaps, the abdominal wall flap is most useful because the procedure can be performed easily, safely, and within a short time.

Fifteen cases underwent split-thickness skin grafting after NPWT, with a 100% survival rate. Although split-thickness skin grafts offer poor color, contour, and texture match at the recipient site and are not as robust as full-thickness skin (potentially blistering or ulcerating more easily if subjected to wear and tear), their advantages include the availability of very large graft areas and improved graft take with thinner grafts (23).

Infection is relatively common in agricultural injuries, with deep wound infections or osteomyelitis occurring in 18% to 19% of these cases (24). Other reports suggest that the infection rate approaches 100% following major limb replantation after traumatic farm injuries (25). A high infection rate was another characteristic of hand injuries caused by corn huskers. Postoperative infection occurred in 13 cases (38.2%), with most infected cases coming from HISS IV injuries, although none of the patients had positive blood cultures. This suggests that the more severe the injury, the higher the infection rate.

As indicated by previous studies, most open-fracture infections were caused by gram-negative rods and gram-positive staphylococci (12). In other research on agricultural injuries, the most commonly isolated microbes following open upper extremity injuries were coagulase-negative Staphylococci (46%), Enterobacter species (42%), Candida species (31%), Stenotrophomonas maltophilia (27%), Staphylococcus aureus (23%), and Pseudomonas aeruginosa (23%) (16). These studies are similar to our results, with a correlation coefficient of 0.923 between the HISS classification and the number of pathogenic bacteria. The reasons for resistance to initial antimicrobial therapy in patients with corn-husker injuries are believed to be: (1) irrational use of antibiotics, including inappropriate dosage and duration; (2) broad-spectrum, empiric antibiotic therapy's inability to effectively target all microbes in open wounds; and (3) prolonged hospitalization increasing the chance of infection. Antimicrobial therapy based on culture and *in vitro* susceptibility data from tissue cultures may be more effective in reducing antibiotic resistance (26). Agricultural hand injuries, particularly those caused by corn-husker equipment, present significant antibiotic resistance challenges. The study identified a predominance (89.5%) of Gram-negative bacilli, with Pseudomonas aeruginosa and Burkholderia cepacia being most common. Staphylococcus aureus demonstrated resistance to tetracyclines, macrolides, and clindamycin, while Gram-negative pathogens showed varied resistance patterns. Standard empiric therapy often proved inadequate, suggesting culture-guided antimicrobial treatment may be more effective. Infection significantly extended hospitalization and increased costs. These findings highlight the importance of targeted antimicrobial strategies based on local resistance patterns in agricultural trauma management.

In our study, 8 patients (47.1%) who underwent NPWT had positive wound cultures. The NPWT group showed a higher infection rate, possibly because the negative pressure tightens wound contact, which could trap bacteria and hinder proper fluid drainage. The incidence of wound infection in the NPWT and No NPWT groups is shown in Table 3, with no significant difference between the two groups. This result may be related to complications of NPWT, such as wound infection due to sponge retention, massive bleeding, infectious erosion of the aorta, and severe soft tissue infection (27).

The reasons for infection are as follows: (1) The main cause was the insertion of corn husks and soil, which carried numerous pathogens, between soft tissue and bone during the injury process. Debridement and irrigation were often insufficient to completely remove these microorganisms. (2) These injuries typically occurred in autumn, a season conducive to bacterial reproduction. (3) The mechanism of corn husker injury, including crushing, avulsion, or friction trauma, decreased the local soft tissue's ability to resist infection, greatly increasing infection rates after debridement. (4) The administration of broad-spectrum, empiric antibiotic therapy has limited effectiveness in treating wounds contaminated by multiple bacteria.

Infection is a significant factor in prolonged hospitalization, leading to increased treatment costs and poor patient outcomes. This suggests that reasonable and effective prevention and control of infection during treatment is key to shortening hospitalization time and reducing medical costs. During an average follow-up of 12 months, we found that postoperative outcomes were often unsatisfactory, frequently due to stiffness secondary to finger joint fractures and infections. Consequently, prevention of occupational injuries is of great importance. Most of these accidents could be prevented with the use of protective clothing, better education, and improved safety precautions (28).

Corn-husker hand injuries exhibit patterns similar to those caused by agricultural machinery across low- and middle-income countries (LMICs). The infection rate in corn-picker hand injuries has been reported to be 15%, which can vary based on crop types such as sugarcane or rice (28). Pathogens implicated in corn-husker hand injuries differ depending on environmental exposure and local conditions in various agricultural settings (28, 29). When comparing treatment between corn-husker hand injuries and other agricultural injuries, microsurgical reconstruction has become a cornerstone in managing complex hand trauma, offering refined options for soft-tissue coverage (29, 30).

Future research should investigate the knowledge and awareness among farmers and their caregivers regarding safety precautions and the use of protective equipment. Machine design improvements should include emergency shutdown mechanisms operating within 0.15 s, physical barriers around snapping rollers, and two-hand operation controls. Retrofittable guards for existing equipment could improve safety without requiring full replacement. Personal protective equipment, including specialized cut-resistant gloves and forearm guards, should be standardized. Conducting relative surveys to evaluate their understanding of injury risks and safety practices can help develop the preventive measures. This baseline data would be benefit to minimize the risk of hand injuries and to improve safety in agriculture.

This study has several key limitations. The retrospective design introduces potential selection bias and limits causal inference. The small sample size significantly constrains statistical power, particularly for subgroup analyses. The single-center scope restricts generalizability to other regions with different agricultural practices. Future research requires multicenter, prospective designs with standardized outcome measures.

## Conclusion

The current study reveals the characteristics of corn-husker equipment hand injuries, including epidemiology, injury mechanisms, surgical methods, and infections. This represents a new type of agriculture-related injury leading to multiple hand traumas, such as soft tissue defects, multiple fractures, and neurovascular injuries. Due to the complexity of these injuries, diverse surgical methods are employed, with NPWT playing an important role in treatment. Effective anti-infective treatment can reduce infection rates, shorten hospital stays, and decrease hospitalization costs, making it one of the most effective approaches. Given the high rate of disability associated with these injuries, prevention and proper use of this type of equipment are crucial in avoiding these serious accidents.

## Data Availability

The raw data supporting the conclusions of this article will be made available by the authors, without undue reservation.

## References

[1] Mucci N Traversini V Lulli LG Baldassarre A Galea RP Arcangeli G. Upper limb’s injuries in agriculture: a systematic review. Int J Environ Res Public Health. (2020) 17(12), 4501. 10.3390/ijerph17124501 32585878 PMC7345507

[2] Yaffe MA Kaplan FT. Agricultural injuries to the hand and upper extremity. J Am Acad Orthop Surg. (2014) 22(10):605–13. 10.5435/JAAOS-22-10-605 25281255

[3] Lower T Mitchell RJ. Farm injury hospitalisations in New South Wales (2010 to 2014). Aust N Z J Public Health. (2017) 41(4):388–93. 10.1111/1753-6405.12686 28712118

[4] Momcilovic D Prokes B Janjic Z. Mechanical cornpicker hand injuries. Med Pregl. (2005) 58(9–10):479–82. 10.2298/MPNS0510479M 16526250

[5] Campbell DA Kay SP. The hand injury severity scoring system. J Hand Surg Br. (1996) 21(3):295–8. 10.1016/S0266-7681(05)80187-1 8771461

[6] Chun S. Management of farm-related injuries to the upper extremity. Hand Clin. (1999) 15(2):201–19, vii. 10.1016/S0749-0712(21)00458-3 10361632

[7] Gupta A Wolff TW. Management of the mangled hand and forearm. J Am Acad Orthop Surg. (1995) 3(4):226–36. 10.5435/00124635-199507000-00005 10795029

[8] Bhardwaj P Sankaran A Sabapathy SR. Skeletal fixation in a mutilated hand. Hand Clin. (2016) 32(4):505–17. 10.1016/j.hcl.2016.06.001 27712751

[9] Cigna E Lo Torto F Maruccia M Ruggieri M Zaccheddu F Chen HC Postoperative care in finger replantation: our case-load and review of the literature. Eur Rev Med Pharmacol Sci. (2015) 19(14):2552–61. 26221881

[10] Vitrat-Hincky V Lebeau B Bozonnet E Falcon D Pradel P Faure O Severe filamentous fungal infections after widespread tissue damage due to traumatic injury: six cases and review of the literature. Scand J Infect Dis. (2009) 41(6–7):491–500. 10.1080/00365540902856537 19353426

[11] Lee J. Efficacy of cultures in the management of open fractures. Clin Orthop Relat Res. (1997) 339:71–5. 10.1097/00003086-199706000-00010 9186203

[12] Forward D Moran CG. Diagnosis and immediate care of open fractures. Hosp Med. (2002) 63(5):298–9. 10.12968/hosp.2002.63.5.2024 12066350

[13] Bailey BN. Skin cover in hand injuries. Injury. (1971) 2(4):294–304. 10.1016/S0020-1383(71)80080-3 4939516

[14] DeRoo LA Rautiainen RH. A systematic review of farm safety interventions. Am J Prev Med. (2000) 18(4 Suppl):51–62. 10.1016/S0749-3797(00)00141-0 10793281

[15] Cogbill TH Steenlage ES Landercasper J Strutt PJ. Death and disability from agricultural injuries in Wisconsin: a 12-year experience with 739 patients. J Trauma. (1991) 31(12):1632–7. 10.1097/00005373-199112000-00012 1749035

[16] Goodman AD Got CJ Weiss AC. Crush injuries of the hand. J Hand Surg Am. (2017) 42(6):456–63. 10.1016/j.jhsa.2017.03.028 28450098

[17] Stannard JP Volgas DA Stewart R McGwin G Jr Alonso JE. Negative pressure wound therapy after severe open fractures: a prospective randomized study. J Orthop Trauma. (2009) 23(8):552–7. 10.1097/BOT.0b013e3181a2e2b6 19704269

[18] Braakenburg A Obdeijn MC Feitz R van Rooij IA van Griethuysen AJ Klinkenbijl JH. The clinical efficacy and cost effectiveness of the vacuum-assisted closure technique in the management of acute and chronic wounds: a randomized controlled trial. Plast Reconstr Surg. (2006) 118(2):390–7; discussion 8−400. 10.1097/01.prs.0000227675.63744.af 16874208

[19] Leininger BE Rasmussen TE Smith DL Jenkins DH Coppola C. Experience with wound VAC and delayed primary closure of contaminated soft tissue injuries in Iraq. J Trauma. (2006) 61(5):1207–11. 10.1097/01.ta.0000241150.15342.da 17099530

[20] Giessler GA Erdmann D Germann G. Soft tissue coverage in devastating hand injuries. Hand Clin. (2003) 19(1):63–71, vi. 10.1016/S0749-0712(02)00128-2 12683447

[21] Erne H Schmauss D Schmauss V Ehrl D. Postoperative negative pressure therapy significantly reduces flap complications in distally based peroneus brevis flaps: experiences from 74 cases. Injury. (2016) 47(6):1288–92. 10.1016/j.injury.2016.02.017 26980646

[22] Zhang Y Wang X Wu Y Li S Zhang D Ma X Grafts vs. flaps: a comparative study of Bracka repair and staged transverse preputial island flap urethroplasty for proximal hypospadias with severe ventral curvature. Front Pediatr. (2023) 11:1214464. 10.3389/fped.2023.1214464 37416816 PMC10321133

[23] Copuroglu C Heybeli N Ozcan M Yilmaz B Ciftdemir M Copuroglu E. Major extremity injuries associated with farmyard accidents. ScientificWorldJournal. (2012) 2012:314038. 10.1100/2012/314038 23002385 PMC3353295

[24] McClure SK Shaughnessy WJ. Farm-related limb amputations in children. J Pediatr Orthop. (2005) 25(2):133–7. 10.1097/01.bpo.0000149864.42777.96 15718888

[25] Ali MH Hoekzema NA Bakleh M Shin AY Osmon DR. The microbiology and risk of infection following open, agricultural upper extremity injuries. J Hand Surg Am. (2008) 33(1):87–93. 10.1016/j.jhsa.2007.09.003 18261671

[26] Saeed MU Kennedy DJ. A retained sponge is a complication of vacuum-assisted closure therapy. Int J Low Extrem Wounds. (2007) 6(3):153–4. 10.1177/1534734607305597 17909174

[27] Walsh M. Farm accidents: their causes and the development of a nurse led accident prevention strategy. Emerg Nurse. (2000) 8(7):24–31. 10.7748/en2000.11.8.7.24.c1340 11935649

[28] Obradović-Tomasev M Jovanović M Vucković N Popović A. Fungal infections in corn picker hand injury. Srp Arh Celok Lek. (2016) 144(1–2):52–5. 10.2298/SARH1602052O 27276858

[29] Gilblom EA Johnson AB Sahr S Sang HI. Using partnerships and multiple data sources to surveil agricultural injuries: considerations and recommendations. J Agromedicine. (2024) 29(2):197–205. 10.1080/1059924X.2023.2293835 38108301 PMC10932915

[30] Adani R Tarallo L Caccese AF Delcroix L Cardin-Langlois E Innocenti M. Microsurgical soft tissue and bone transfers in complex hand trauma. Clin Plast Surg. (2014) 41(3):361–83. 10.1016/j.cps.2014.03.002 24996459

